# Prevalence and risk indicators for severe periodontitis in Côte d’Ivoire

**DOI:** 10.34172/japid.2022.008

**Published:** 2022-05-31

**Authors:** Nadin Thérèse Koffi-Coulibaly, Zocko Ange Désiré Pockpa, Gnaba Samson Mobio, Xavier Struillou, Assem Soueidan

**Affiliations:** ^1^Department of Periodontology, Dental College, Felix Houphouet Boigny University, Cote d’Ivoire; ^2^Department of Periodontology, Faculty of Dental Surgery, University of Nantes, France

**Keywords:** Periodontitis, prevalence, severity, attachment loss, risk indicators, Côte d'Ivoire

## Abstract

**Background:**

The present study evaluated the prevalence of severe periodontitis (SP) and determined the possible relevant risk factors among patients referred to the Periodontology Department at the Dental Care Center of the Odontostomatology Training and Research Unit of Abidjan, in Côte d’Ivoire.

**Methods:**

This retrospective observational study was based on 1087 patients data aged 18‒80 years, who were treated in the periodontology department from December 2008 to December 2018. Severe periodontitis (stages III or IV) was defined as interdental clinical attachment loss (CAL)>5 mm at two non-adjacent teeth. Two groups were considered: patients with severe periodontitis (test) or without severe periodontitis (control). Differences between the two groups were tested using the chi-squared test and ANOVA. Furthermore, logistic regression analysis was used to model the relationship between the severity of periodontitis and covariables as potential risk indicators.

**Results:**

43.4% of patients had severe periodontitis with a mean CAL of 6.89 mm. SP was associated with age (*P*=0.004), socioeconomic status (*P*=0.005), smoking habits (*P*=0.000), brushing frequency (*P*=0.000), the number of mobile teeth (*P*<0.001), and the number of lost teeth (*P*<0.001). Logistic regression analysis showed that having at least five mobile teeth (OR= 4.11, 95% CI: 2.95‒5.73) and/or five missing teeth (OR=2.60, 95% CI: 1.85‒3.66) were independent risk indicators for severe periodontal disease.

**Conclusion:**

This Ivorian sample presented a high prevalence of severe periodontal diseases. Therefore, proper public health measures would allow early detection, with targeted and effective treatment of the Ivorian population.

## Introduction

 Periodontitis is a chronic multifactorial inflammatory disease associated with dysbiotic plaque biofilms and characterized by progressive destruction of the tooth-supporting apparatus. Clinical signs include gingival bleeding, clinical attachment loss (CAL), increased probing pocket depths, and radiographically assessed alveolar bone loss.^1^ The progression and the severity of periodontitis are modulated by local, behavioral, and general risk factors (inadequate oral hygiene, socioeconomic status, stress, smoking, alcohol consumption, obesity, and diabetes).^1,2^ If untreated, the course of the disease can lead to severe periodontitis (SP) with severe loss of supporting structures resulting in substantial tooth loss and contributing to systemic inflammation.^3,4^ Severe periodontitis and associated tooth loss have implications for the oral health-related quality of life and general health (masticatory deficit, undernutrition, diabetes, cancers, cardiovascular diseases, and adverse pregnancy outcomes), with psychosocial disabilities (aesthetic deficit, loss of self-esteem) and significant costs related to treatments.^3,5-8^ Epidemiological studies have demonstrated considerable variations in prevalence, incidence, and risk indicators of severe periodontitis between regions and countries. Differences could be attributed to variations in periodontal examination protocols and case definitions between studies.^9^ In 2010, severe periodontitis was the sixth most prevalent condition, affecting 10.8% (95% UI: 10.1‒11.6%) of the general adult population or 743 million people worldwide. Between 1990 and 2010, the global age-standardized prevalence of SP was estimated at 11.2% (95% UI: 10.4‒11.9% in 1990 and 10.5‒12.0% in 2010). This review used three comparable quantitative indicators of severe periodontitis: CPITN class 4 (PD≥6 mm), CAL>6 mm, and PD>5 mm).^3^ Using the Center for Disease Control and the American Academy of Periodontology (CDC-AAP) proposed case definitions of periodontitis for population-based surveys,^10^ the prevalence of severe periodontitis in adults was estimated at 7.8%, in the USA,^11^ and 29% in Brazil.^12^ According to the EFP-AAP classification for clinical practice,^13,1^ the frequency of subjects with severe periodontitis (stages III and IV) characterized by CAL≥5 mm, was 54% in Turkey,^14^ and >30% in China.^15^ In West Africa, the prevalence of subjects with generalized stage IV grade C periodontitis was 50.4% in Senegal.^16^ To the best of our knowledge, no epidemiological study is available on the prevalence and risk factors of severe periodontitis in Côte d’Ivoire. However, preventing and/or treating this disease remains a problem faced by general practitioners in Côte d’Ivoire due to late consultation by patients and the lack of data about this destructive pathology with a negative impact on the quality of life. Therefore, this study aimed to evaluate the prevalence of severe periodontitis (stages III and IV) and determine the possible related risk factors in patients referred to the Periodontology Department at the Dental Care Center of the Odontostomatology Training and Research Unit of Abidjan, Côte d’Ivoire.

## Methods

###  Study design and settings 

 This retrospective observational study used a subset of data from 1087 patients aged 18‒80 years treated in the Periodontology Department at the Dental Care Center of the Odontostomatology Training and Research Unit of Abidjan. The data were collected from the medical files of patients with periodontitis treated and monitored from December 2008 to December 2018 (10 years) using a survey sheet. Fully completed files of patients treated for periodontitis only during this period were included in this study. Clinical files of patients with gingivitis and periodontitis as manifestations of systemic diseases or necrotizing periodontal diseases were excluded from the study. The anonymity of information included in clinical files was guaranteed. Indeed, prior anonymization work was carried out by one author (KCN) between the initial clinical examination sheets for each patient and those used for this study. The study protocol was approved by the Scientific and Ethical Committee of the Odontostomatology Training and Research Unit, University Félix Houphouët Boigny of Abidjan (approval number 381/18).

###  Data Collection and Diagnostic Criteria

 In practice, in the Periodontology Department, patients are treated by postgraduate students under the supervision of a senior periodontist. All newly referred patients are interviewed, followed by full-mouth periodontal and radiographic examinations. During the periodontal examination, all permanent fully erupted teeth were evaluated manually, excluding third molars, using William’s periodontal probe (Michigan O probe, Hu-Friedy Mfg. Co., Chicago, IL, USA). The data collected included sociodemographic variables (age, gender, socioeconomic status, life, and oral habits) and clinical variables, including plaque index (PI) (Silness and Löe),^17^bleeding on probing (BOP) (Mühlemann and son, 1971),^18^ number of missing teeth, number of mobile teeth, probing depth (PD), gingival recession (REC) and clinical attachment loss (CAL). PI and BOP were recorded at six sites on each tooth (mesiobuccal, mid-buccal, distobuccal, mesiolingual, mid-lingual, and distolingual). At the same sites, PD, REC, and CAL were measured. PD was measured as the distance from the free gingival margin to the bottom of the pocket, and REC as the distance from the cementoenamel junction to the free gingival margin. CAL was calculated as the sum of PD and REC. Routinely, all data is collected on examination sheets and is archived in each patient’s file. From the data we already had in our database for 10 years, for each patient, two authors (PZ) performed a new diagnostic workup according to the new criteria of the 2018 EFP/AAP. Indeed, in this study, periodontitis was diagnosed according to the clinical criteria established in the 2018 EFP/AAP new classification of Periodontal and Peri-Implant Diseases and Conditions.^1,13^ This classification is based on the stages and grades of periodontitis. In the present study, only stages were considered. Periodontitis severity staging was defined by the interproximal CAL at sites with the greatest attachment loss: a CAL of 1–2 mm was defined as Stage I (mild periodontitis), of 3–4 mm as Stage II (moderate periodontitis), and of ≥5 mm at two non-adjacent teeth as Stages III–IV (severe periodontitis). Then, for this study, the subjects were assigned to two groups according to the mean value of the clinical attachment loss: the non-severe periodontitis (NSP) group (i.e., mild and moderate periodontitis combined: CAL<5 mm) and the severe periodontitis (SP) group (CAL≥5 mm).

###  Statistical analysis

 Statistical analysis was performed using SPSS 22.0 (SPSS Inc., Chicago, IL, USA). Descriptive statistics included the calculation of mean values and standard deviations for quantitative variables. Categorical variables were expressed as frequencies and percentages. Differences between the individuals with and without SP were tested using chi-squared test and ANOVA. A P-value of <0.05 was considered statistically significant. Furthermore, logistic regression analysis was used to model the relationship between the severity of periodontitis and covariables as potential risk indicators.

## Results

Table 1 shows the distribution of sociodemographic, lifestyle and oral hygiene habits data in the studied sample according to severity of the periodontitis. 1087 patients, aged 18-80 year were selected for this study including 615 (56.6%) cases of non-severe periodontitis (stages I and II) and 472 (43.4%) cases of severe periodontitis (stages III and IV). The mean age of the sample was 37.86 years (±14.26); the 18-34 age group (35.23%) was the most represented. The majority of patients were male (59.0%), had a low socioeconomic status (65.8%), did not smoke (73.9%) or did not drunk alcohol (81.7%) and brushed their teeth more than twice a day (56.5%). Severe periodontitis were significantly associated with age (P=0.004), socioeconomic status (P=0.005), smoking habits (P<0.001), brushing frequency (P<0.001). No significant association were obtained with gender (P=0.771) and alcool status (P=0.183).

**Table 1:** Socio-demographic characteristics, lifestyle and oral hygiene habits of the sample according to the severity of periodontitis (N = 1087)

	**NSP (Stage I/II)**	**SP (Stage III/IV)**	**Total sample**	**p-value**
**Variables**	**n (%)**	**n (%)**	**N (%)**	
**Age (years)**				0.004*
18-34	286 (51.8)	266 (48.2)	552 (50.8)	
35-49	182 (62.5)	109 (37.5)	291 (26.8)	
50-64	105 (57.4)	78 (42.6)	183 (16.8)	
65-80	42 (68.9)	19 (31.1)	61 (05.6)	
**Sex**				0.771
Male	365 (56.9)	276 (43.1)	641 (59.0)	
Female	250 (56.1)	196 (43.9)	446 (41.0)	
**Socio-economic status**				0.005*
Low	383 (53.6)	332 (46.4)	715 (65.8)	
High	232 (62.4)	140 (37.6)	372 (34.2)	
**Smoking habits**				< 0.001*
No	544 (67.7)	259 (32.3)	803 (73.9)	
Yes	71 (25.0)	213 (75.0)	284 (26.1)	
**Alcool status**				0.183
No	494 (55.6)	394 (44.4)	888 (81.7)	
Yes	121 (60.8)	78 (39.2)	199 (18.3)	
**Brushing frequency**				< 0.001*
< 2	1 (0.2)	472 (99.8)	473 (43.5)	
≥ 2	614 (100.0)	0 (0.0)	614 (56.5)	
**Total**	615 (56.6)	472 (43.4)	1087 (100)	

NSP: Non-Severe Periodontitis; SP: Severe Periodontitis. *p < 0.05

**Figure 1:**
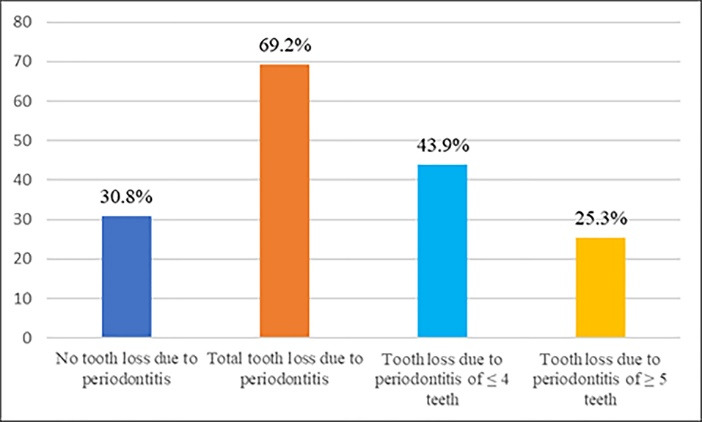
Prevalence and severity of tooth loss due to periodontitis

 The periodontal status of the patients is described in Table 2. 43.4% of the sample had a SP with a mean CAL value of 6.89±2.36 mm. Compared to patients with NSP, patients with SP had almost the same amount of plaque (2.01±0.74 vs. 1.94±0.73) and inflammation (1.76±0.70 vs. 1.74±0.81), had deeper periodontal pockets (6,73±1.12 vs. 4.03±0.82), higher gingival recessions (3.34±0.44 mm vs. 0.2±0.71 mm), greater mean attachment loss (9.1±1.66 mm vs 4.8±0.83 mm), more mobile teeth (5.38±6.04 vs. 1.49±3.49) and more missing teeth (4.35±4.41 vs. 2.58±3.60). These observations were statistically significant for PD (P<0.001), REC (P<0.001), CAL (P<0.001), number of mobile teeth (P<0.001) and teeth lost (P<0.001). Prevalence and severity of tooth loss due to periodontitis are presented in figure 1. Two-third (69.2%) of patients had at least one missing tooth: 43.9% had lost 1-4 teeth and 25.3% ≥5 teeth.

**Table 2:** Periodontal status of the sample according to the severity of periodontitis (N = 1087)

**Variable**	**NSP (Stage I/II)** **Mean (SD)**	**SP (Stage III/IV)** **Mean (SD)**	**Total sample** **Mean (SD) **	**p-value**
**PI**	1.94 (± 0.73)	2.01 (± 0.74)	1.97 (± 0.74)	0.123
**BOP**	1.74 (± 0.81)	1.76 (± 0.70)	1.75 (± 0.77)	0.681
**PD**	4.03 (± 0.82)	6.73 (± 1.12)	5.32 (± 1.06)	< 0.001*
**REC**	0.24 (± 0.71)	3.34 (± 0.44)	1.59 (± 1.89)	< 0.001*
**CAL**	4.84 (± 0.83)	9.17 (± 1.66)	6.89 (± 2.36)	< 0.001*
**Teeth lost**	2.58 (± 3.60)	4.35 (± 4.41)	3.35 (± 4.07)	< 0.001*
**Mobile teeth**	1.49 (± 3.49)	5.38 (± 6.04)	3.18 (± 5.14)	< 0.001*
**Total**	615 (56.6%)	472 (43.4%)	1087 (100%)	

SD: standard deviation; PI: Plaque index; BOP: Bleeding on probing; PD probing depth; REC: recession; CAL: clinical attachment loss. *p < 0.05

**Figure 2:**
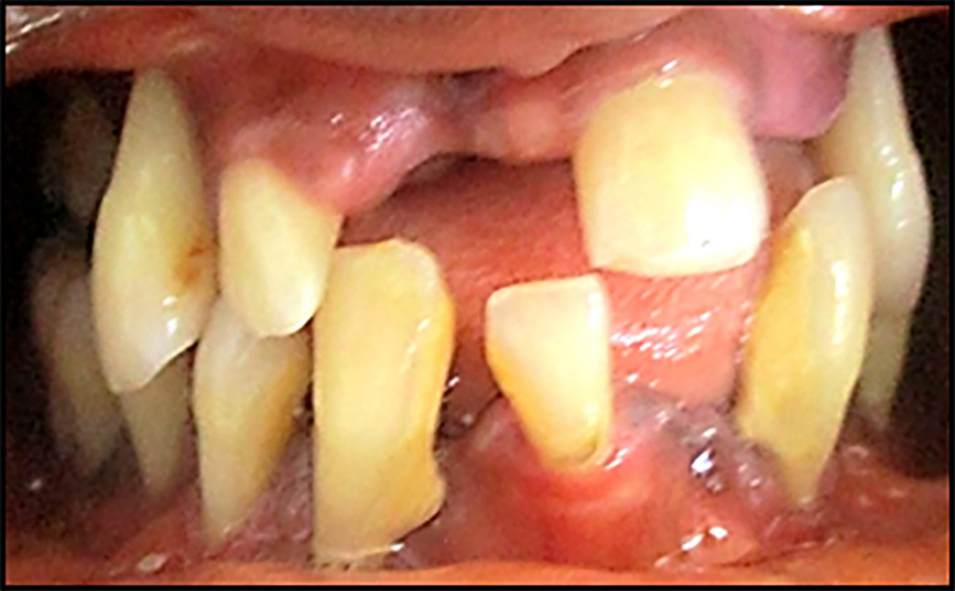
Clinical front view of generalized severe periodontitis (Stage IV / Grade C) of a 18 year patient. Note the presence of attachment loss, dental migration/mobility, and missing teeth: destruction exceeds expectation given biofilm deposits.

**Figure 3:**
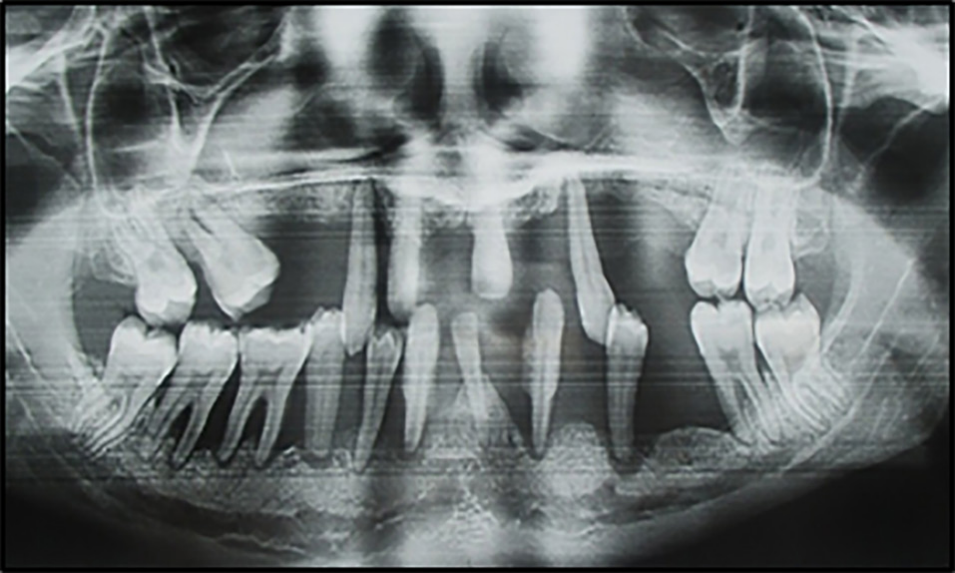
A panoramic X-ray of the patient presented in figure 1. Note severe generalized radiographic bone loss extending to the apical third of the root: % bone loss/age (100% / 18 = 5.5) > 1.0

 The significant variables selected for analysis by logistic regression, shown in Table 3, were: variables not related to periodontal status: age, socioeconomic status, smoking habits, brushing frequency and variables related to periodontal status: teeth lost and mobile teeth. Having at least 5 mobile teeth (OR= 4.11, 95% CI: 2.95‒5.73, P=0.000), and/or 5 missing teeth (OR=2.60, 95% CI: 1.85‒3.66, P=0.000) were identified as independent risk indicators for severe periodontitis (Figures 2 and 3).

**Table 3:** Logistic regression for independent variables with the occurrence of SP (CAL ≥ 5mm)

**Variable**	**SP (Stage III/IV) (N= 472)**		
	**OR**	**95% CI**	**p-value**
**Teeth lost**			
<5	1		< 0.001*
≥ 5	2.60	[1.85-3.66]	
**Mobile teeth**			
<5	1		< 0.001*
≥ 5	4.11	[2.95-5.73]	

OR: Odds Ratio; CI: 95% Confidence Intervals *p < 0.05

## Discussion

 In this study, the prevalence of severe periodontitis (stages III and IV) and risk indicators have been evaluated among patients referred to the periodontology department at the dental care center of training and research unit of odontostomatology in Abidjan, the economic capital of Côte d’Ivoire. The outcomes show that 43.4% of the patients presents severe periodontitis. This prevalence is higher than those reported in several other countries.^11,12,19^ This can be due that epidemiological studies used different periodontal recording protocols, case deﬁnitions and surveyed different age groups.^9^ In the present study, a full- mouth clinical evaluation (6 sites per tooth) was performed and SP was confirmed when interdental CAL was ≥5 mm at two non-adjacent teeth. The definition of SP varies among studies which represents a real obstacle for the comparison of results between them. In adult Kiriri Indians from Northeast Brazil (2011), SP was observed in 29% of the 215 individuals.^12^ SP cases were identified in individuals with≥2 interproximal sites with CAL≥6 mm, not on the same tooth and ≥ 1 proximal site with PD≥5 mm, according to the CDC-AAP proposed case definitions of severe periodontitis.^10^ In Spain (2008‒2011), the prevalence of severe periodontitis deﬁned as Community Periodontal Index (CPI)=4, was 10.1%.^20^ In France (2002-2003), 19.7% of individuals had severe periodontitis, confirmed in subjects with CAL>5 mm.^19^ Based on the EFP-AAP classification for clinical practice,^1,13^ the frequency of subjects with SP (stages III and IV) characterized by CAL≥5 mm, was more than 30% in China.^15^ However, the prevalence of SP in our study was similar to those reported in some African countries: 40.2% in South Africa,^21^ 44.3% in Uganda^22^ and 50.4% in Senegal.^16^ In addition to the methodological heterogeneity, this various prevalence between studies could be due to inequalities in socioeconomic conditions, access to dental services and other factors.^2,5^ In this sample, 65.8% of patients had a low socioeconomic status; and SP was significantly more frequent in the low socioeconomic category than in the high socioeconomic category (46.4% vs. 37.6%; P=0.005). The impact of socioeconomic status on periodontitis has been demonstrated in previous studies, subjects with the lowest-income and education levels being at the highest risk.^23,24,7^In many low income populations, consultations are often late in an advanced stage of the disease, mainly due to a lack of financial resources and inequalities in access to oral health care. As a result, patients were often referred by ther practionner to periodontists, at a public dental care center with affordable rates, for the treatment of complex cases of periodontal disease.^12,16,22^ Furthermore, SP was significantly associated with age (p= 0.004). The significant association between age and loss of attachment has been reported by previous studies which consistently show an increase of the prevalence with increasing age among adults over 30 years of age in Caucasian populations.^19,20,25,11^In the present study, SP was significantly more frequent in the youngest age group (18-34 years). In the 1999 classification,^26^ “aggressive periodontitis” were described as a severe and rapidly progressing periodontitis, characterized by a rapid attachment loss and alveolar bone destruction, that occurs in young individuals, more frequently in Africa.^27,28^ The severe attachment loss resulted in high rates of ≥5 mobile teeth and ≥ 5 missing teeth, identified as independent risk indicators for severe periodontitis. Patients with severe periodontitis had significantly more mobile teeth (5.38±6.04 vs. 1.49±3.49) and more missing teeth (4.35±4.41 vs. 2.58±3.60) than patients with non-severe periodontitis. In low-income countries, apart from spontaneous tooth loss due to late consultations, tooth extraction is the most common treatment, due to the lack of adequate technical facilities.^12,29^ Periodontitis was more frequent in men (59.20%) than in women (40.80%). A male predominance of 63.8% and 56.4% was also found in Senegal^16^and in the United States,^10^ respectively. However, some studies have shown a female predominance.^16^ Regarding oral hygiene habits and behavioural factors, SP was associated with brushing frequency (P<0.001) and smoking habits (P<0.001). The majority of patients who brushed their teeth less than twice a day (99.8%) had SP. Compared to non-smokers, active smokers had significantly higher prevalence of SP (75.0% vs. 25.0%; P<0.001). Smoking is a risk factors associated with the severity of periodontitis.^1^

 The present study had some limitations. The clinical examinations were performed by several students under the supervision of periodontist, and the intra-examiners reproducibility has not been determined. Our study was retrospective, and the data recorded on bone loss, tooth loss and complexity variables was not always complete. Therefore, the analysis of periodontitis severity staging was only based on the mean interproximal CAL; therefore the difference between stages III and IV was not possible and the prevalence of SP could be underestimated.

## Conclusions

 Within the limitations of the study, the results showed a high prevalence of severe periodontitis in the population evaluated. Factors of age, male gender, smoke tobacco, and low brushing frequency may significantly increase the risk for severe periodontitis. Having at least 5 mobile teeth and/or 5 missing teeth constitutes independent risk indicators for severe periodontitis in Ivorians. The outcomes may also have clinical implications. It may be concluded that in Côte d’Ivoire, a person who is a regular smoker, who brushes less than twice a day, presenting many mobiles or missing teeth, have a high risk for severe periodontitis. Appropriate public health measures would allow early detection and targeted and effective treatment of the Ivorian population.

## Authors’ Contributions

 KCN, MG contributed to conception, design. PZ contributed to data acquisition, analysis. KCN, PZ, AS, XS, MG contributed to data interpretation drafted, and critically revised the article. All authors gave their final approval and agree to be accountable for all aspects of the work.

## Funding

 There was no funding support for the study.

## Availability of data

 The datasets used and/or analyzed during the current study are available from the corresponding author on reasonable request.

## Ethics Approval

 The research project was approved by the Scientific and Ethical Committee of the Odontostomatology Training and Research Unit, University Félix Houphouët Boigny of Abidjan (approval number 381/18).

## Competing Interests

 The authors declare no conflict of interest related to the study.
